# Contamination of sulfonamide antibiotics and sulfamethazine-resistant bacteria in the downstream and estuarine areas of Jiulong River in Southeast China

**DOI:** 10.1007/s11356-015-4473-z

**Published:** 2015-04-16

**Authors:** Danyun Ou, Bin Chen, Renao Bai, Puqing Song, Heshan Lin

**Affiliations:** Third Institute of Oceanography, State Oceanic Administration, 178 Daxue Road, Xiamen, 361005 China

**Keywords:** Sulfonamides, Sulfamethazine-resistant bacteria, Bacterial diversity, Drug resistance, Surface water, Southeast China

## Abstract

Surface water samples from downstream and estuarine areas of Jiulong River were collected in August 2011 and May 2012 for detecting sulfonamide antibiotic residues and isolating sulfamethazine-resistant bacteria. Sulfamethazine was detected in all samples in May 2012 at an average concentration of 78.3 ng L^−1^, which was the highest among the nine sulfonamide antibiotics determined. Sulfamethazine-resistant bacteria (SRB) were screened using antibiotic-containing agar plates. The SRB average abundance in the samples was 3.69 × 10^4^ and 2.17 × 10^3^ CFUs mL^−1^ in August 2011 and May 2012, respectively, and was positively correlated to sulfamethazine concentration in May 2012. The 16S rRNA gene sequencing of all the 121 SRB isolates revealed high diversity. Furthermore, the SRB isolates exhibited multidrug resistance, with 48.7 % showing resistance to at least three antibiotics. The abundance and persistence of highly diverse SRB and their multidrug resistance are likely to demonstrate the transferable pressure from coastal environments on public health.

**Electronic supplementary material:**

The online version of this article (doi:10.1007/s11356-015-4473-z) contains supplementary material, which is available to authorized users.

## Introduction

The occurrence of antibiotics in aquatic environments is of ecotoxicological concern because of potential ecosystem alteration. Prolonged exposure to low doses of antibiotics leads to selective proliferation of resistant bacteria, which could transfer antimicrobial resistance genes to other bacterial species (Dantas et al. 2008; Levy and Marshall 2004) with difficult-to-predict consequences on human health (Martínez 2008). The potential of these low antibiotic concentrations to promote antimicrobial resistance has been substantiated by the detection of several antibiotic-resistant bacteria and/or genes in aquaculture sites (Hoa et al. 2011), municipal wastewater discharges (Schwartz et al. 2003; Volkmann et al. 2004), lake water (Czekalski et al. 2012), drinking water (Schwartz et al. 2003), sediment (Pei et al. 2006), and soil environments (Dantas et al. 2008; Hargrave et al. 2008). Aquatic ecosystems have been proposed as reservoirs of antimicrobial resistance (Biyela et al. 2004), and resistant bacteria in coastal environments can represent a serious environmental issue and a means for the spread and evolution of antibiotic-resistant bacteria (Young 1993), thus drawing increasing concern in China.

Sulfonamides are a big family of sulfonamide-related antibiotics commonly used in clinical and veterinary medicine and have drawn increasing attention of researchers in China and elsewhere (Gutiérrez et al. 2010; Pinna et al. 2012; Zhu et al. 2013). A number of studies have shown that sulfonamide residues are widespread in the coastal aquatic environment and may pose high ecological risk to estuaries, such as Bohai Bay (Zhang et al. 2013a), Laizhou Bay (Zhang et al. 2012), Beibu Gulf (Zheng et al. 2012), and so on (Zou et al. 2011). In the environment, sulfonamides can bind to dihydropteroate synthase (DHPS) in the bacterium and inhibit dihydrofolic acid formation which is essential for bacterial growth. Sulfonamide resistance in bacteria is mediated by the horizontal transfer of the foreign *dhps* gene or a part of it or by means of plasmids which carry sul-genes encoding alternative drug-resistant variants of the DHPS enzyme (Sköld 2000). Positive relationship between the number of sulfonamide-resistant bacteria and the concentrations of sulfonamides was examined indicating that sulfonamides could induce bacteria to generate corresponding resistance (Na et al. 2014). Sulfonamides could inhibit the bacterial enzyme activity and cause a relative community shift towards gram-negative bacteria and towards an increased proportion of fungal biomass in soil (Gutiérrez et al. 2010; Pinna et al. 2012).

Jiulong River is the second largest river in the Fujian Province, China; this latter province comprising a population of more than five million people. High concentration of antibiotics has been detected in this watershed (Chen et al. 2013; Zheng et al. 2011). In the present study, we characterized the pattern of contamination by measuring the sulfonamide residues and sulfamethazine-resistant bacteria (SRB) in the downstream and estuarine areas of Jiulong River during the rainy and dry seasons. Furthermore, the diversity and multidrug resistance of SRB were also studied. We aimed to draw public attention on the consequence of antibiotics contamination by evaluating the impact of sulfonamides residual on aquatic bacterial community based on the occurrence of sulfonamide-resistant bacteria and their antimicrobial resistance. The results presented here are conspicuous since previous studies addressing impact of sulfonamides on aquatic microbial parameters failed to highlight any significant effect of the sulfonamide on microbial abundance and diversity.

## Material and methods

### Sample collection

**Fig. 1:**
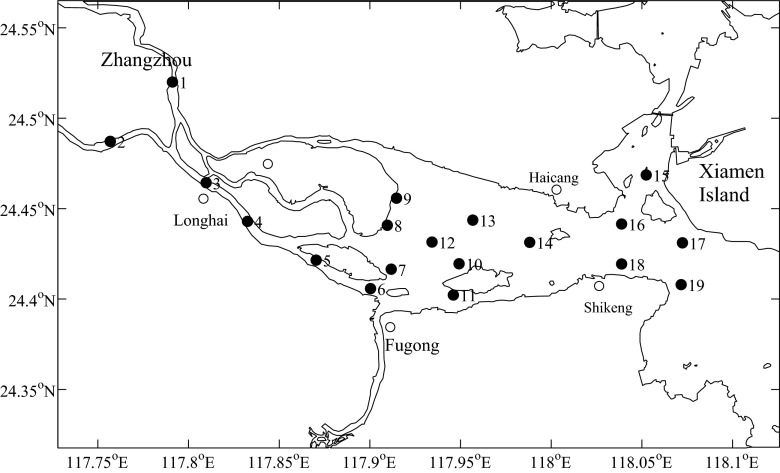
Sampling sites in the downstream and estuarine areas of Jiulong River

Surface water samples were collected along the downstream and estuarine areas of Jiulong River in August 2011 and May 2012, and the sampling sites are indicated in Fig. 1. For antibiotic analysis, 1 L of each aqueous sample was individually collected in 1 N hydrochloric acid pre-treated glass bottles. Simultaneously, for SRB enumeration, 0.5 L of the aqueous sample was individually collected in sterile bags (Nasco, Fort Atkinson, WI, USA). All the samples were put into an ice-packed cooler and transported back to the laboratory for immediate processing.

### Sulfonamide antibiotics analysis

The sulfonamide antibiotics in the water samples were analyzed using ultra-performance liquid chromatography coupled with electrospray tandem mass spectrometry (UPLC–MS-MS) (Su et al. 2007). Standard samples of 14 sulfonamides, namely, sulfamethoxazole, sulfamethizole, sulfapyridine, sulfathiazole, sulfachloropyridazine, sulfadimethoxine, sulfamonomethoxine, sulfamethoxydiazine, sulfamethazine, sulfadiazine, sulfisomidine, sulfacetamide, sulfisoxazole, and sulfadoxin, were purchased from Sigma-Aldrich Co. (St. Louis, MO, USA).

The water samples were filtered through a mixed cellulose ester filter (GE, Piscataway, NJ, USA), 0.2 g of edetate disodium and 100 ng of recovery indicator ^13^C_3_-caffein were added to the filtered water samples and the pH was adjusted to 3.0. The treated water samples were extracted with SPE devices (Sigma-Aldrich Co.) installed with a HLB column (6 mL, 500 mg, Waters, Milford, MA, USA) at a speed of <10 mL min^−1^. The HLB column was pretreated with 6 mL of methanol and 6 mL of acidified purified water (pH = 3.0). After extraction, the bound component was eluted with 6 mL of methanol and flushed with nitrogen gas, and then 60 % methanol was added to make the volume up to 1 mL and the liquid was filtered through a 0.2-μm filter (Whatman, Piscataway, NJ, USA). The blank (2 L of purified water) and recovery indicator (dissolved in 100 mL of seawater) were also subjected to the same procedure. Qualitative and quantitative analyses were performed on a Waters Acquity™ Ultra Performance LC (UPLC) (Waters, Milford, MA, USA) and mass spectrometer Quattro Premier XE MICROMASS (Waters), with an electrospray tandem mass spectrometer (ESI-MS-MS) using multi-reactive monitoring mode.

### Enumeration of SRB

The SRB were enumerated by selective cultivation in marine agar 2216E plates (Difco, BD, Franklin Lakes, NJ, USA) supplemented with 350 μg mL^−1^ sulfamethazine. The water samples were serially diluted with axenic seawater and 100 μL of each dilution were spread onto each plate. *Escherichia coli* was spread to agar amended with and without sulfamethazine for the validation of the sulfamethazine-supplement agar. All the dilutions were prepared in triplicate and the plates were incubated at 28 °C for 6–7 days. A bacterial abundance between 30 and 300 CFUs per plate was used to estimate the abundance of bacteria in the samples, and the percentage of SRB was calibrated with total bacteria enumerated on non-selective 2216E agar plates (Gao et al. 2012b).

### SRB diversity analysis

The SRB colonies were picked up individually and reinoculated into marine 2216E liquid medium and cultured at 28 °C for 1 day. The total DNA was extracted using a bacterial DNA extraction kit (Omega, Norcross, GA, USA) and employed as a template to amplify the 16S rDNA gene with bacterial primers 27f and 1492r (Frank et al. 2008). The PCR products were sequenced (Shenggong Ltd., Shanghai, China) and sequence information was generated using MOTHUR (Schloss et al. 2009). The sequences were blasted against the EZTaxon library to identify the classification information of SRB isolates (Kim et al. 2012).

### Antimicrobial resistance of SRB

The antimicrobial resistance test was conducted using the agar disc diffusion susceptibility test in accordance with the guidelines in the CLSI document M02-A11 and related supplement (Clinical and Laboratory Standards Institute 2009a, b). The disc diffusion test was performed for each SRB isolate using un-supplemented Mueller-Hinton agar and standard antibiotic susceptibility test discs. The antibiotic content in the disc was as follows: tetracycline (TC, 30 μg, tetracycline group), chloramphenicol (CHL, 30 μg, phenicols group), florfenicol (FFC, 75 μg, phenicols group), norfloxacin (NFX, 10 μg, fluoroquinolones group), ampicillin (AMP, 10 μg, penicillins group), benzenesulfonamide (BSF, 300 μg, folate pathway inhibitors group), erythromycin (ETR, 15 μg, macrolides group), or nalidixic acid (NDA, 30 μg, quinolones group). As QC control, a non-resistant *E. coli* isolate was employed. Following incubation for 18–20 h at 35 °C, the diameters of significant inhibition zones around each disc were measured, and the antimicrobial resistance was determined in accordance with the CLSI standard.

## Results

### Detection of sulfonamides

**Fig. 2:**
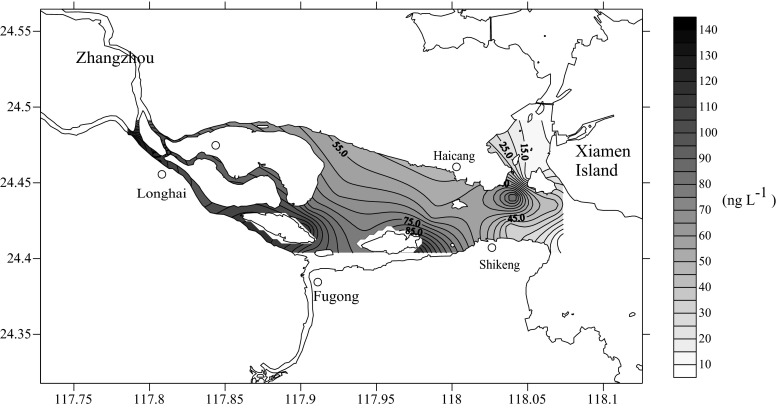
Concentration of sulfamethazine in the surface water of the studied sites

A significant seasonality was observed with regard to the sulfonamide concentration in the downstream and estuarine areas of Jiulong River, as shown in Table 1. The average concentrations of sulfamethoxazole, sulfachloropyridazine, and sulfadiazine were 1.15, 1.72, and 2.34 ng L^−1^ in August 2011, whereas they were 22.13, 25.85, and 22.06 ng L^−1^ in May 2012, respectively. Among the nine sulfonamides detected, sulfamethazine exhibited the highest concentration and detection frequency, with a concentration of 0–53.41 and 4.84–138.66 ng L^−1^ and a detection rate of 16.67 and 100 % in August 2011 and May 2012, respectively. The average concentrations of sulfapyridine, sulfamonomethoxine, sulfamethoxydiazine, sulfacetamide, and sulfadoxin were 3.43, 2.29, 2.29, 7.92, and 38.51 ng L^−1^ in May 2012, respectively, while these sulfonamides were below the limit of detection in August 2011. Furthermore, the concentration of sulfamethazine was higher in the upstream region than in the estuarine area of Jiulong River in May 2012 (Fig. 2), indicating that the upstream region could serve as the reservoir of sulfonamides residual in the Jiulong River.

**Table 1:** Sulfonamide antibiotics content in the surface water of the studied sites

Sulfonamide antibiotics	MDL (ng L^−1^)	Percentage recovery (%)	RSD%	August 2011			May 2012		
				Range of detected value (ng L^−1^)	Average content (ng L^−1^)	Detection frequency	Range of detected value (ng L^−1^)	Average content (ng L^−1^)	Detection frequency
Sulfamethoxazole	0.5	113.66	5.74	2.79–13.02	1.15	16.70 %	7.50–38.05	22.13	100 %
Sulfamethizole	0.1	87.63	5.16	ND	0	0	ND	0	0
Sulfapyridine	0.5	95.36	10.07	ND	0	0	0.91–17.99	3.43	100 %
Sulfathiazole	0.5	99.18	5.47	ND	0	0	ND	0	0
Sulfachloropyridazine	0.1	112.97	5.89	4.71–17.35	1.72	16.70 %	3.02–51.54	25.85	100 %
Sulfadimethoxine	0.1	102.88	4.46	ND	0	0	ND	0	0
Sulfamonomethoxine	0.5	90.83	2.62	ND	0	0	0.31–9.00	2.29	100 %
Sulfamethoxydiazine	0.1	94.91	3.19	ND	0	0	0.31–9.07	2.29	100 %
Sulfamethazine	0.5	116.73	4.03	16.91–53.41	5.53	16.70 %	4.84–138.66	78.31	100 %
Sulfadiazine	1	100.21	2.63	7.59–25.06	2.34	16.70 %	1.71–40.78	22.06	100 %
Sulfisomidine	0.2	91.94	3.74	ND	0	0	ND	0	0
Sulfacetamide	0.5	100.59	3.83	ND	0	0	5.35–26.17	7.92	77.80 %
Sulfisoxazole	0.1	100.10	3.09	ND	0	0	ND	0	0
Sulfadoxin	0.5	122.47	7.23	ND	0	0	3.79–63.18	38.51	100 %

*MDL* method detection limit, *RSD* relative standard deviation, *percentage recovery* percentage of standard antibiotics concentration detected to the concentration that started with, *ND* not detected

### Occurrence of SRB

**Fig. 3:**
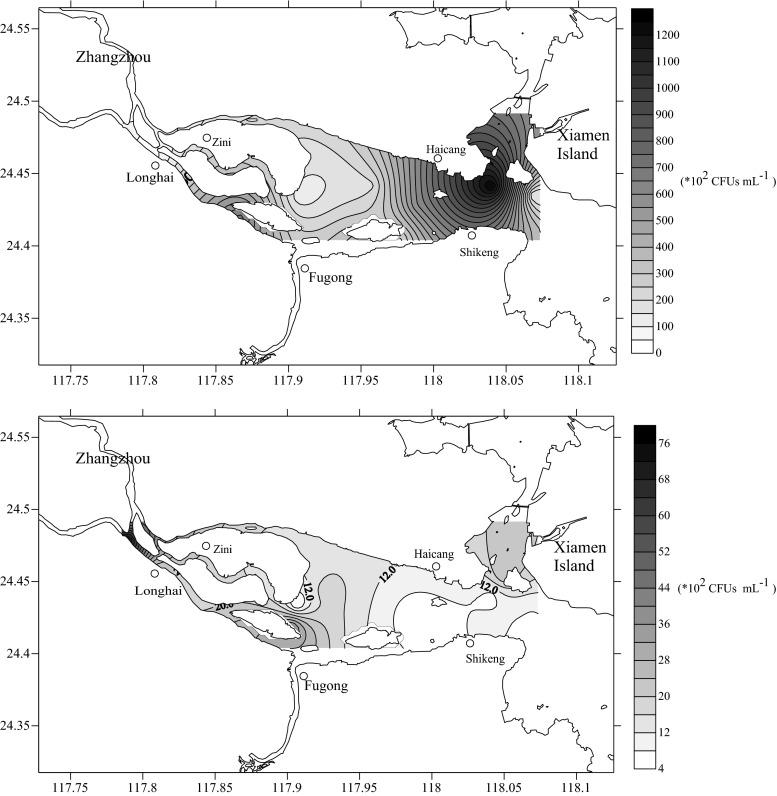
Distribution of SRB in the surface water of the studied sites in August 2011 (*top*) and May 2012 (*bottom*)

Using the same samples employed for sulfonamide antibiotic detection, the culturable bacteria and SRB were enumerated using 2216 marine broth agar and sulfamethazine-supplemented 2216 marine broth agar, respectively. The abundance of SRB peaked to 1.26 × 10^5^ CFUs mL^−1^ near the estuarine area in August 2011, while high abundance of SRB occurred in the upstream area of Jiulong River in May 2012 (Fig. 3, Supplemental Table 2). The average abundances of SRB and total culturable bacteria were 3.69 × 10^4^ and 1.83 × 10^5^ CFUs mL^−1^ in August 2011, respectively, which were 13.5 and 1.1 times higher than those noted in May 2012. The abundance of SRB showed almost the same seasonal trend as that of the total culturable bacteria; i.e., the total abundance of culturable bacteria and SRB in the entire study area was higher in August 2011 than that in May 2012, with the exception of sites 1 and 2. The occurrence rates of SRB, as reflected by the ratio of abundance of SRB to total culturable bacteria, were 2.55–39.90 % in August 2011 and 0.65–23.0 % in May 2012.

### Diversity of SRB

**Fig. 4:**
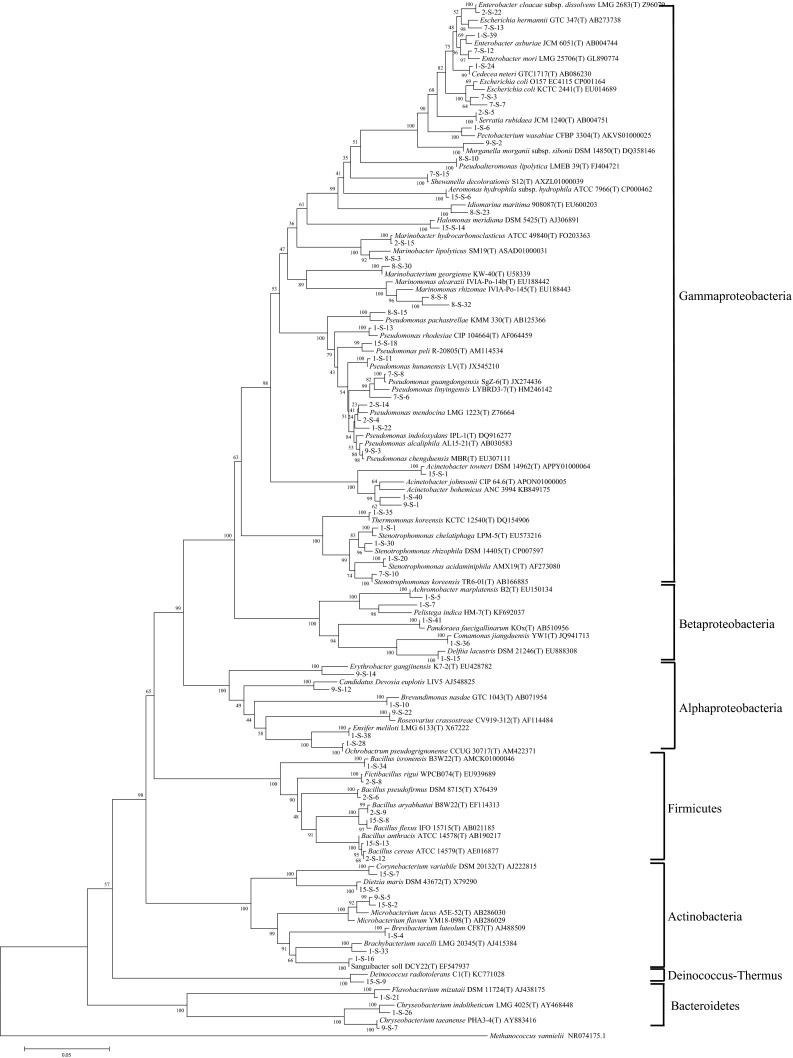
Phylogenetic tree of the SRB isolated from Jiulong River based on 16S rDNA sequences using UPGMA method with the Kimura 2-parameter model for nucleotide change. The *branch distances* represent nucleotide substitution rate and the *scale bar* represents the expected number of changes per homologous nucleotide position. The *first number* in the names of the SRB isolates indicates the sampling site

In total, 121 SRB isolates (67 from August 2011 and 52 from May 2012) from sites 1, 2, 7, 8, 9, and 15, representing the predominant sulfonamide-resistant bacteria in Jiulong River during the sampling period, were selected for further phylogenetic analysis. The coverage of the SRB isolates based on their 16S ribosomal RNA (rRNA) gene was 69.1 % as the cutoff value was set to 0.03, indicating that the sequence number of the SRB isolates was adequate to reflect the 16S rRNA gene diversity of SRB in Jiulong River. The Shannon–Weiner index based on 16S sequences of the SRB community was around 2.40 with a cutoff value of 0.03. Most of the SRB isolates (113 isolates) showed 97 % or higher similarity with their best-matched sequences as reviewed in Supplement Table 3. The SRB in Jiulong River were quite diverse, with at least 40 genera and 67 species, and the phylogenetic tree based on 16S rDNA sequences further confirmed this finding (Fig. 4). A total of 67 SRB species belonged to five phyla of Proteobacteria (49), Actinobacteria (7), Firmicutes (7), Bacteroidetes (3), and Deinococcus-Thermus (1). Among all the eight classes identified, most of the SRB belonged to Gamma-Proteobacteria (38 species), which were found in all of the six sampling sites. Among the 67 SRB species detected, *Pseudomonas* spp. were the most abundant, and, altogether, ten *Pseudomonas* spp. (*P. alcaliphila*, *P. chengduensis*, *P. guangdongensis*, *P. hunanensis*, *P. indoloxydans*, *P. linyingensis*, *P. mendocina*, *P. pachastrellae*, *P. peli*, and *P. rhodesiaei*) were recovered, followed by *Bacillus* spp. (six species), *Stenotrophomonas* spp. (four species), and *Acinetobacter* spp. (three species). Besides the common estuarine and marine species, SRB isolates affiliated to Enterobacteriaceae such as *E. coli*, *Enterobacter cloacae*, and *Serratia rubidaea* were also detected in the study area, with some of them being potentially pathogenic to humans. In addition, the SRB isolates identified in our study showed no significant difference between the two sampling seasons at all the classification levels.

### Antimicrobial resistance of SRB

The antimicrobial resistance profile of the 39 SRB isolates (belonged to 25 genera, 39 species) against eight antimicrobial agents divided in seven groups were reviewed in Table 2. Among the eight antimicrobial agents used for drug resistance analysis, SRB showed high resistance to AMP (89.7 %), medium resistance to BSF (61.5 %), FFC (38.5 %), NDA (38.5 %), CHL (35.9 %), and TC (33.3 %), and mild resistance to ETR (28.2 %) and NFX (15.4 %). A total of 16 drug-resistance patterns (one single-drug, three two-drug, one three-drug, three four-drug, two five-drug, three six-drug, two seven-drug and one eight-drug) were observed for the 39 SRB isolates. Among these, 12 patterns represented multidrug resistance (resistant to antimicrobial agents from at least three groups) and was observed from 19 SRB isolates belonged to 15 genera (Table 2). Among the 39 SRB isolates tested, several species from potential pathogen groups were found to inhibited multidrug resistance. For instance, 3 of 6 *Pseudomonas* spp. tested showed resistance to 3–5 antimicrobial agents, 2 *Acinetobacter* spp. were resistant to 4 antimicrobial agents, and 2 *Stenotrophomonas* spp. inhibited resistance to 4 and 6 antimicrobial agents, respectively. These results indicated that bacteria in coastal waterbodies had become a reservoir of multidrug resistance which deserve additional attention for the public health.

**Table 2:** Antimicrobial resistance profile of 39 SRB isolates from the Jiulong River

SRB isolates^a^	Identification	TC	CHL	FFC	NFX	AMP	BSF	ETR	NDA
1-S-40	*Acinetobacter johnsonii*	R	S	S	S	R	R	S	R
1-S-31	*Acinetobacter towneri*	S	S	S	S	R	S	I	S
1-S-2	*Acinetobacter towneri*	R	I	I	S	R	R	R	S
1-S-8	*Aeromonas hydrophila*	S	S	S	S	R	S	I	S
2-S-9	*Bacillus aryabhattai*	S	S	S	S	R	S	S	S
2-S-12	*Bacillus cereus*	S	S	S	S	R	R	S	S
1-S-34	*Bacillus isronensis*	S	S	S	S	R	R	S	I
2-S-6	*Bacillus pseudofirmus*	S	S	S	S	R	S	S	S
1-S-33	*Brachybacterium sacelli*	S	R	R	S	R	R	R	S
1-S-4	*Brevibacterium luteolum*	R	R	R	R	R	R	I	R
1-S-10	*Brevundimonas nasdae*	S	R	R	R	R	R	I	R
1-S-24	*Cedecea neteri*	S	S	S	S	R	S	I	S
1-S-26	*Chryseobacterium indoltheticum*	R	R	R	I	R	R	I	R
1-S-36	*Comamonas jiangduensis*	R	R	R	R	S	R	I	R
1-S-15	*Delftia lacustris*	S	S	S	S	R	S	I	S
1-S-39	*Enterobacter asburiae*	S	S	S	S	R	S	I	S
2-S-22	*Enterobacter cloacae*	R	R	R	I	R	R	I	R
2-S-8	*Fictibacillus rigui*	R	R	R	I	R	R	R	R
1-S-21	*Flavobacterium mizutaii*	R	R	R	R	R	R	I	R
8-s-23	*Idiomarina maritima*	S	S	S	S	R	S	I	S
2-S-15	*Marinobacter hydrocarbonoclasticus*	R	R	R	I	R	R	I	R
8-s-3	*Marinobacter lipolyticus*	S	S	S	S	R	S	S	S
8-s-30	*Marinobacterium georgiense*	S	S	S	S	R	S	I	S
1-S-43	*Nocardioides insulae*	S	S	S	S	S	S	S	S
1-S-28	*Ochrobactrum pseudogrignonense*	S	S	S	S	R	R	I	S
1-S-41	*Pandoraea faecigallinarum*	R	R	R	R	R	R	R	R
1-S-6	*Pectobacterium wasabiae*	S	S	S	S	S	S	I	S
1-S-7	*Pelistega indica*	R	R	S	R	R	R	R	R
1-S-22	*Pseudomonas alcaliphila*	S	S	S	S	R	S	I	I
1-S-11	*Pseudomonas hunanensis*	S	R	R	S	R	S	R	R
2-S-14	*Pseudomonas indoloxydans*	S	S	S	S	S	R	S	R
2-S-4	*Pseudomonas mendocina*	S	I	R	S	R	S	R	S
8-s-15	*Pseudomonas pachastrellae*	S	I	R	I	R	R	I	R
1-S-13	*Pseudomonas rhodesiae*	S	S	S	S	R	S	I	S
1-S-16	*Sanguibacter soli*	S	R	R	I	R	R	R	S
2-S-1	*Stenotrophomonas chelatiphaga*	R	R	R	I	R	R	I	R
1-S-1	*Stenotrophomonas chelatiphaga*	S	I	S	S	R	S	R	S
1-S-19	*Stenotrophomonas maltophilia*	R	S	S	I	R	R	R	I
1-S-35	*Thermomonas koreensis*	S	S	S	S	R	S	R	S

*R* resistance, *I* intermediate, *S* susceptible

^a^First number in the name of SRB isolates indicated the sampling sites

## Discussion

Sulfonamides are synthetic antimicrobials, and hence, their detection in aquatic environments is not expected from natural sources. In China, sulfonamides are still in use in poultry and aquaculture due to their low price, although they are gradually being replaced by β-lactams, macrolides, and other antibiotics (Zou et al. 2011). Among the 14 sulfonamides analyzed in the present study, nine (sulfamethoxazole, sulfapyridine, sulfachloropyridazine, sulfamonomethoxine, sulfamethoxydiazine, sulfamethazine, sulfadiazine, sulfacetamide, and sulfadoxin) were detected in the surface water of Jiulong river with a detection frequency of 77.8–100 % at the sampling sites. Among these, sulfamethazine showed the highest average concentration of 78.31 ng L^−1^, followed by sulfadoxin (35.81 ng L^−1^). It must be noted that, to date, high concentrations of sulfonamides have never been reported in Jiulong River, and the concentrations of sulfonamides observed in the present study are comparable with those reported in the Bohai Bay in China and are far higher than those noted in the Pearl River, coast of Yellow Sea in China, and German Bight (Bendz et al. 2005; Cheng et al. 2014; Na et al. 2013; Xu et al. 2007; Zou et al. 2011). Sulfonamides have a high potential to resist degradation, which can explain their high rate in aquatic environments (Xu et al. 2007; Zou et al. 2011). In the present study, the contents of sulfonamides detected in May 2012 were higher than those noted in August 2011, which may be due to higher photolysis rate in the summer season (Boreen et al. 2004) or decreased assumption of sulfonamides. Meanwhile, the overall content of sulfonamides in the surface water of Jiulong River is generally higher than that of tetracycline and florfenicol (Ou et al. 2013). Previous study has suggested that sulfamethazine might serve as a marker of livestock-source contamination (Baquero et al. 2008); hence, it is reasonable to speculate that livestock might be the most possible source of sulfamethazine contamination in Jiulong River for the they largely contributed to the nutrient input into Jiulong River (Li et al. 2011). An earlier study on the distribution of antimicrobial resistance genes revealed the positive relationship between *sulII* gene encoding for sulfonamide resistance and nutrient levels (Zhang et al. 2013b).

Development of resistance to antibiotics is a global environmental problem, and evidence had clearly shown that the coastal water bodies are potential reservoirs of antibiotic-resistant organisms and associated genes (Hoa et al. 2011; Zhang et al. 2013a). At very low concentrations, antibiotics might act as signaling hormones to stimulate the proliferation of some microorganisms, and it appears that such alterations in the microbial ecosystems, especially selection of some resistant pathogenic organisms, might be a huge threat to public health (Baquero et al. 2008). In the present study, the overall abundance and the ratio of sulfamethazine-resistant bacteria vs. total culturable bacteria were generally high in the coastal water of Jiulong River, with values ranging from 267 to 126,400 CFUs mL^−1^ and from 0.65 to 39.9 %, respectively, in the 19 sampled sites during August 2011 and May 2012. While the incidence of SRB was high in Jiulong River across the two sampling seasons, at some sites, the sulfonamide concentrations were lower. This may due to the persistence of antibiotic-resistant bacteria (Antunes et al. 2005; Bean et al. 2005) for genetic linkage of sulfonamide resistance to other resistance determinants, and horizontal gene transfer may also contribute to the dissemination of sulfonamide-resistant bacteria in the environments (Bean et al. 2005; Enne et al. 2001). It must be noted that reports on the enumeration of SRB in the seawater has been limited.

Statistical analysis revealed a positive correlation between the occurrence rates of SRB (ratio of SRB to culturable bacteria) and sulfamethazine concentration in all sampling sites (*p* = 0.035) in May 2012, while no significant correlation was observed in August 2011. A similar correlation tendency was also found between the occurrence rates of SRB and total concentration of nine sulfonamides. Meanwhile, SRB abundance and sulfamethazine content showed almost the opposite seasonal tendency, this may be due to the fact that the higher photolysis rate in the summer season might have degraded sulfamethazine, but their previous effect might still be mirrored in a high abundance of resistant bacteria. Nevertheless, some studies have also confirmed the weak relationship between sulfonamide contamination and resistant bacteria, which may largely be due to the persistence of sulfonamide resistance genes from gene transfer and endogenous origin from plasmids (Hoa et al. 2011; Sköld 2000).

Besides the persistence of SRB, the high diversity of SRB isolates revealed in the present study also signified potential threat of SRB in Jiulong River. A diverse array of SRB isolates were obtained, with 16S rDNA sequencing indicating 67 different species, including opportunistic pathogens and indigenous marine bacteria. Among these, *Acinetobacter* spp., *Bacillus* spp., and *Pseudomonas* spp. had been previously reported to be intrinsically resistant to sulfonamides, while some isolates had been affiliated with groups of bacteria that have been poorly characterized in terms of sulfonamide resistance, such as *Marinobacter hydrocarbonoclasticus*, *Marinobacter lipolyticus*, *Marinomonas alcarazii*, *Marinomonas rhizomae*, *Serratia rubidaea*, and bacteria affiliated with Alphaproteobacteria, Betaproteobacteria, and Deinococcus-Thermus. This result suggested that sulfonamide resistance is very common in the aquatic environment of Jiulong River and that diverse bacteria had developed resistance to sulfonamides.

Apart from resistance to sulfonamide, the SRB isolates were also found to possess multidrug resistance, with 80 % of the SRB isolates showing resistance to at least three antibiotics, indicating that these bacterial strains could potentially be serving as a reservoir for diverse antimicrobial resistance genes. Phenotyping of antibiotic-resistant SRB isolates showed that the most common resistance pattern was OZC/AMP/CD, which accounted for 20.5 % of the 39 isolates, followed by TC/CHL/FFC/OZC/AMP/CD/BSF/NDA (10.3 %). It should be noted that among the ten antibiotic agents tested, the resistances for AMP, OZC, and CD were the most frequent, even though these isolates were selected by sulfamethazine, suggesting that a single antibiotic has the potential to co-select for diverse resistances (Fernández-Alarcón et al. 2010). Sulfonamides are still in used in aquaculture in China due to their low price (Gao et al. 2012a), thus public health can potentially be affected by the use of antibiotics in aquaculture or agriculture.

The coastal water environment has a direct and intimate contact with human life and may transport antibiotics and antimicrobial-resistant bacteria to animals inhabited around, posing threats to public health (Ding and He 2010). Previous studies have shown an alteration in the composition of the microbial community and an increase in resistance genes (*sulI* and *sulII*) and sulfonamide-resistant bacterial count following exposure to sulfonamides (Demoling et al. 2009; Gutiérrez et al. 2010; Hammesfahr et al. 2008; Kotzerke et al. 2008; Pinna et al. 2012; Schauss et al. 2009). The occurrence and persistence of highly diverse SRB and their multidrug resistance observed in the present study suggested the transferable pressure from coastal environments on public health. To assess this potential risk, future studies should focus on the ability of different antibiotics used in aquatic environments to co-select multiple resistances and determine whether the genes conferring resistance can be transferred to other bacteria, including those of human-health concern.

## Conclusions

Surface water samples from the Jiulong River in southeast China were analyzed for 14 sulfonamides residues and sulfamethazine-resistant bacteria. Among nine sulfonamides detected, sulfamethazine showed the highest content and positive ratio. The recovered resistant bacteria were enumerated, and their number was found to be positively correlated with sulfamethazine concentration in one sampling season. The persistence of highly diverse SRB and their multidrug resistance demonstrate the effects of antibiotic contamination on the bacterial community and potential threat to public health.

## Electronic supplementary material

Supplement material Table 1 (DOCX 22 kb)

Supplement material Table 2 (DOCX 18 kb)

Supplement material Table 3 (DOCX 36 kb)
