# Supplementary material for: Contamination of sulfonamide antibiotics and sulfamethazine-resistant bacteria in the downstream and estuarine areas of Jiulong River in Southeast China
Source: Environ Sci Pollut Res Int. 2015 Apr 16;22(16):12104–13. doi: 10.1007/s11356-015-4473-z (PMC4515247; doi:10.1007/s11356-015-4473-z)
Supplement: Supplementary file 1 — (DOCX 22 kb) [file 11356_2015_4473_MOESM1_ESM.docx]

Supplement material

Tab. 1-1 Sampling records on August 20^th^, 2011

| Date:  Agu. 20^th^, 2011 | | Sampling ship:  MinLongYu 62191 | | Weather condition*:  Cloudy, wind≤3, 26-34 ℃ | |
| --- | --- | --- | --- | --- | --- |
| Site | Sampling time | Longitude | Latitude | Water temperature | Salinity |
| 1 | 16:52 | 117°47'31.02'' | 24°31'09.11'' | 31.4 | 0.0 |
| 2 | 16:44 | 117°45'27.67'' | 24°29'10.89'' | 31.4 | 0.0 |
| 3 | 16:28 | 117°48'37.83'' | 24°27'48.66'' | 31.7 | 0.5 |
| 4 | 16:09 | 117°50'00.07'' | 24°26'31.57'' | 31.7 | 5.8 |
| 5 | 15:51 | 117°52'16.26'' | 24°25'14.48'' | 32.0 | 10.8 |
| 7 | 15:30 | 117°54'45.29'' | 24°24'56.48'' | 31.0 | 15.7 |
| 8 | 14:09 | 117°54'37.6'' | 24°26'23.86'' | 31.7 | 16.3 |
| 9 | 14:24 | 117°54'55.82'' | 24°27'17.82'' | 30.3 | 23.9 |
| 10 | 13:52 | 117°57'00.07'' | 24°25'07.28'' | 32.2 | 11.8 |
| 11 | 13:26 | 117°56'48.66'' | 24°24'05.1'' | 31.1 | 17.7 |
| 12 | 13:09 | 117°56'06.29'' | 24°25'50.48'' | 30.2 | 23.9 |
| 13 | 12:23 | 117°57'27.2'' | 24°26'34.14'' | 31.7 | 16.4 |
| 14 | 10:02 | 117°59'20.27'' | 24°25'49.96'' | 30.4 | 19.1 |
| 15 | 8:25 | 118°03'11.55'' | 24°28'04.09'' | 29.0 | 30.2 |
| 16 | 8:40 | 118°02'22.73'' | 24°26'26.44'' | 29.7 | 30.4 |
| 17 | 8:53 | 118°04'23.5'' | 24°25'48.9'' | 28.6 | 30.6 |
| 18 | 9:32 | 118°02'22.73'' | 24°25'06.77'' | 29.5 | 24.6 |
| 19 | 9:07 | 118°04'20.93'' | 24°24'25.65'' | 28.9 | 28.3 |

* weather condition could be recovered at http://lishi.tianqi.com/xiamen/201108.html

Tab. 1-2 Sampling records on May 21^st^, 2012

| Date:  May 21^st^, 2012 | | Sampling ship:  MinXiamenDu 1175 | | Weather condition*:  Cloudy, wind≤3, 20-27 ℃ | |
| --- | --- | --- | --- | --- | --- |
| Site | Sampling time | Longitude | Latitude | Water temperature | Salinity |
| 1 | 11:53 | 117°47'05.02" | 24°30'10.12" | 23.7 | 0.0 |
| 2 | 11:40 | 117°46'25.68" | 24°29′10.89″ | 24.3 | 0.0 |
| 3 | 11:24 | 117°48'15.84" | 24°27'59.67" | 24.4 | 0.0 |
| 4 | 11:02 | 117°50′0.07″ | 24°26'05.57" | 24.7 | 0.0 |
| 5 | 10:40 | 117°52′16.26″ | 24°25'02.48" | 24.6 | 0.5 |
| 6 | 10:23 | 117°54′4.2″ | 24°24′17.94″ | 24.8 | 4.4 |
| 7 | 10:10 | 117°54'18.29" | 24°24′56.48″ | 24.7 | 5.8 |
| 8 | 13:26 | 117°54′37.60″ | 24°26′23.86″ | 24.6 | 7.7 |
| 9 | 13:37 | 117°55'10.83" | 24°27′17.82″ | 24.3 | 14.9 |
| 10 | 9:53 | 117°56'30.07" | 24°24'48.28" | 25.1 | 7.3 |
| 11 | 9:45 | 117°56′48.66″ | 24°24′5.1″ | 25.1 | 12.0 |
| 12 | 13:50 | 117°56′6.29″ | 24°25′50.48″ | 24.7 | 6.4 |
| 14 | 16:02 | 117°59′20.27″ | 24°25′49.96″ | 24.8 | 14.8 |
| 15 | 7:46 | 118°3′11.55″ | 24°28′4.09″ | 24.8 | 24.9 |
| 16 | 8:07 | 118°02'42.73" | 24°26'20.45" | 24.9 | 10.7 |
| 17 | 8:26 | 118°4′23.50″ | 24°25′48.90″ | 24.8 | 15.3 |
| 18 | 9:00 | 118°2′22.73″ | 24°25′6.77″ | 24.9 | 18.8 |
| 19 | 8:42 | 118°4′20.93″ | 24°24′25.65″ | 24.6 | 28.4 |

* weather condition could be recovered at http://lishi.tianqi.com/xiamen/201205.html
