# Supplementary material for: Contamination of sulfonamide antibiotics and sulfamethazine-resistant bacteria in the downstream and estuarine areas of Jiulong River in Southeast China
Source: Environ Sci Pollut Res Int. 2015 Apr 16;22(16):12104–13. doi: 10.1007/s11356-015-4473-z (PMC4515247; doi:10.1007/s11356-015-4473-z)
Supplement: Supplementary file 2 — (DOCX 18 kb) [file 11356_2015_4473_MOESM2_ESM.docx]

Tab. 2 Occurrence of SRB in the surface water of Jiulong River

(The results show the average values of triplicates)

| Site | August 2011 | | | May 2012 | | |
| --- | --- | --- | --- | --- | --- | --- |
|  | Abundance of cultivable bacteria (cells mL^-1^) | Abundance of SRB (cells mL^-1^) | Ratio of SRB | Abundance of cultivable bacteria (cells mL^-1^) | Abundance of SRB (cells mL^-1^) | Ratio of SRB |
| 1 | 13,000 | 3750 | 28.85% | 75,333 | 3800 | 5.04% |
| 2 | 26,000 | 6350 | 24.42% | 33,333 | 7733 | 23.20% |
| 3 | 103,800 | 2650 | 2.55% | 77,333 | 2247 | 2.91% |
| 4 | NA | NA | NA | 73,333 | 1800 | 2.45% |
| 5 | 207,500 | 68,200 | 32.87% | 226,667 | 2533 | 1.12% |
| 6 | NA | NA | NA | 200,000 | 2800 | 1.40% |
| 7 | 127,500 | 29,500 | 23.14% | 34,000 | 2800 | 8.24% |
| 8 | 213,500 | 11,920 | 5.58% | 41,333 | 267 | 0.65% |
| 9 | NA | NA | NA | 43,333 | 1867 | 4.31% |
| 10 | 399,500 | 23,120 | 5.79% | 46,667 | 3933 | 8.43% |
| 11 | 154,000 | 37,400 | 24.29% | 40,667 | 1400 | 3.44% |
| 12 | 85,000 | 23,000 | 27.06% | 26,000 | 833 | 3.21% |
| 13 | 53,000 | 18,950 | 35.75% | NA | NA | NA |
| 14 | 146,000 | 58,250 | 39.90% | 19,667 | 500 | 2.54% |
| 15 | 420,000 | 95,800 | 22.81% | 240,000 | 2400 | 1.00% |
| 16 | 487,000 | 126,400 | 25.95% | 66,667 | 600 | 0.90% |
| 17 | 245,000 | 14,140 | 5.77% | 120,000 | 867 | 0.72% |
| 18 | NA | NA | NA | 146,667 | 1000 | 0.68% |
| 19 | 90,000 | 34,000 | 37.78% | 54,667 | 667 | 1.22% |
| Average | 184,720 | 36,895 | 22.83% | 86,981 | 2114 | 3.97% |

NA: Samples not available due to construction program of local bridge.
