# Supplementary material for: Contamination of sulfonamide antibiotics and sulfamethazine-resistant bacteria in the downstream and estuarine areas of Jiulong River in Southeast China
Source: Environ Sci Pollut Res Int. 2015 Apr 16;22(16):12104–13. doi: 10.1007/s11356-015-4473-z (PMC4515247; doi:10.1007/s11356-015-4473-z)
Supplement: Supplementary file 3 — (DOCX 36 kb) [file 11356_2015_4473_MOESM3_ESM.docx]

Tab. 3 reviewed of blast results of SRB isolates using EzBio on line bacterial identification tool (<http://www.ezbiocloud.net/eztaxon/identify>)

| **Number** | **Query** | **closest match** | **similarity%** | **completence%** | **accession** |
| --- | --- | --- | --- | --- | --- |
| SRB1 | 1-S-1 | Stenotrophomonas chelatiphaga LPM-5(T) | 99.47 | 89.2 | EU573216 |
| SRB2 | 1-S-2 | Acinetobacter towneri DSM 14962(T) | 98.85 | 89.8 | APPY01000064 |
| SRB3 | 1-S-4 | Brevibacterium luteolum CF87(T) | 99.92 | 90.4 | AJ488509 |
| SRB4 | 1-S-5 | Achromobacter marplatensis B2(T) | 99.69 | 90.2 | EU150134 |
| SRB5 | 1-S-6 | Pectobacterium wasabiae CFBP 3304(T) | 98.7 | 89.4 | AKVS01000025 |
| SRB6 | 1-S-7 | Pelistega indica HM-7(T) | 96.02 | 89.7 | KF692037 |
| SRB7 | 1-S-8 | Aeromonas hydrophila subsp. hydrophila ATCC 7966(T) | 99.85 | 89.3 | CP000462 |
| SRB8 | 1-S-10 | Brevundimonas nasdae GTC 1043(T) | 99.54 | 94.7 | AB071954 |
| SRB9 | 1-S-11 | Pseudomonas hunanensis' LV(T) | 99.85 | 89.8 | JX545210 |
| SRB10 | 1-S-13 | Pseudomonas rhodesiae CIP 104664(T) | 99.39 | 89.8 | AF064459 |
| SRB11 | 1-S-14 | Pelistega indica HM-7(T) | 96.02 | 89.6 | KF692037 |
| SRB12 | 1-S-15 | Delftia lacustris DSM 21246(T) | 99.77 | 90 | EU888308 |
| SRB13 | 1-S-16 | Sanguibacter soli DCY22(T) | 99.85 | 90.8 | EF547937 |
| SRB14 | 1-S-17 | Pelistega indica HM-7(T) | 96.02 | 89.7 | KF692037 |
| SRB15 | 1-S-20 | Stenotrophomonas acidaminiphila AMX19(T) | 99.69 | 89.2 | AF273080 |
| SRB16 | 1-S-21 | Flavobacterium mizutaii DSM 11724(T) | 98.93 | 90.3 | AJ438175 |
| SRB17 | 1-S-22 | Pseudomonas alcaliphila AL15-21(T) | 98.7 | 89.8 | AB030583 |
| SRB18 | 1-S-23 | Castellaniella daejeonensis MJ06(T) | 47.38 | 47.6 | GQ241321 |
| SRB19 | 1-S-24 | Cedecea neteri GTC1717(T) | 99.85 | 89.5 | AB086230 |
| SRB20 | 1-S-25 | Pseudoalteromonas lipolytica LMEB 39(T) | 100 | 89.5 | FJ404721 |
| SRB21 | 1-S-26 | Chryseobacterium indoltheticum LMG 4025(T) | 98.55 | 90.8 | AY468448 |
| SRB22 | 1-S-28 | Ochrobactrum pseudogrignonense CCUG 30717(T) | 100 | 93.3 | AM422371 |
| SRB23 | 1-S-30 | Stenotrophomonas rhizophila DSM 14405(T) | 99.16 | 89.3 | CP007597 |
| SRB24 | 1-S-31 | Acinetobacter towneri DSM 14962(T) | 99.16 | 89.7 | APPY01000064 |
| SRB25 | 1-S-32 | Flavobacterium mizutaii DSM 11724(T) | 98.93 | 90.3 | AJ438175 |
| SRB26 | 1-S-33 | Brachybacterium sacelli LMG 20345(T) | 98.62 | 90.9 | AJ415384 |
| SRB27 | 1-S-34 | Bacillus isronensis B3W22(T) | 100 | 88.6 | AMCK01000046 |
| SRB28 | 1-S-35 | Thermomonas koreensis KCTC 12540(T) | 100 | 89.3 | DQ154906 |
| SRB29 | 1-S-36 | Comamonas jiangduensis YW1(T) | 99.85 | 90 | JQ941713 |
| SRB30 | 1-S-37 | Comamonas jiangduensis YW1(T) | 98.93 | 90 | JQ941713 |
| SRB31 | 1-S-38 | Ensifer meliloti LMG 6133(T) | 99.92 | 93.1 | X67222 |
| SRB32 | 1-S-39 | Enterobacter asburiae JCM 6051(T) | 99.16 | 89.6 | AB004744 |
| SRB33 | 1-S-40 | Acinetobacter johnsonii CIP 64.6(T) | 97.4 | 89.7 | APON01000005 |
| SRB34 | 1-S-41 | Pandoraea faecigallinarum KOx(T) | 99.77 | 90 | AB510956 |
| SRB35 | 1-S-42 | Sanguibacter soli DCY22(T) | 97.48 | 90.8 | EF547937 |
| SRB36 | 1-S-43 | Nocardioides insulae DS-51(T) | 48.21 | 48.2 | DQ786794 |
| SRB37 | 1-S-45 | Brachybacterium sacelli LMG 20345(T) | 98.6 | 90.8 | AJ415384 |
| SRB38 | 2-S-1 | Serratia rubidaea JCM 1240(T) | 100 | 89.5 | AB004751 |
| SRB39 | 2-S-2 | Serratia rubidaea JCM 1240(T) | 100 | 89.5 | AB004751 |
| SRB40 | 2-S-4 | Pseudomonas mendocina LMG 1223(T) | 99.23 | 89.7 | Z76664 |
| SRB41 | 2-S-5 | Serratia rubidaea JCM 1240(T) | 100 | 89.5 | AB004751 |
| SRB42 | 2-S-6 | Bacillus pseudofirmus DSM 8715(T) | 99.77 | 88.9 | X76439 |
| SRB43 | 2-S-7 | Serratia rubidaea JCM 1240(T) | 99.92 | 89.5 | AB004751 |
| SRB44 | 2-S-8 | Fictibacillus rigui WPCB074(T) | 99.85 | 89.1 | EU939689 |
| SRB45 | 2-S-9 | Bacillus aryabhattai B8W22(T) | 100 | 88.8 | EF114313 |
| SRB46 | 2-S-10 | Serratia rubidaea JCM 1240(T) | 100 | 89.5 | AB004751 |
| SRB47 | 2-S-11 | Serratia rubidaea JCM 1240(T) | 100 | 89.5 | AB004751 |
| SRB48 | 2-S-12 | Bacillus cereus ATCC 14579(T) | 99.92 | 88.9 | AE016877 |
| SRB49 | 2-S-14 | 'Pseudomonas indoloxydans' IPL-1(T) | 99.08 | 89.9 | DQ916277 |
| SRB50 | 2-S-15 | Marinobacter hydrocarbonoclasticus ATCC 49840(T) | 99.85 | 89.5 | FO203363 |
| SRB51 | 7-S-13 | Escherichia hermannii GTC 347(T) | 98.93 | 89.5 | AB273738 |
| SRB52 | 7-S-12 | Enterobacter mori LMG 25706(T) | 99.31 | 89.5 | GL890774 |
| SRB53 | 7-S-10 | Stenotrophomonas koreensis TR6-01(T) | 99.77 | 89.3 | AB166885 |
| SRB54 | 7-S-8 | Pseudomonas guangdongensis SgZ-6(T) | 99.85 | 89.8 | JX274436 |
| SRB55 | 7-S-7 | Escherichia coli KCTC 2441(T) | 99.23 | 89.3 | EU014689 |
| SRB56 | 7-S-6 | 'Pseudomonas linyingensis' LYBRD3-7(T) | 97.79 | 89.8 | HM246142 |
| SRB57 | 7-S-5 | Escherichia coli O157 EC4115 | 98.62 | 89.2 | CP001164 |
| SRB58 | 2-S-22 | Enterobacter cloacae subsp. dissolvens LMG 2683(T) | 99.77 | 89.6 | Z96079 |
| SRB59 | 2-S-21 | Enterobacter cloacae subsp. dissolvens LMG 2683(T) | 99.77 | 89.6 | Z96079 |
| SRB60 | 2-S-20 | Serratia rubidaea JCM 1240(T) | 100 | 89.5 | AB004751 |
| SRB61 | 7-S-14 | 'Aeromonas dhakensis' LMG 19562(T) | 99.77 | 89.3 | AJ508765 |
| SRB62 | 7-S-15 | Shewanella decolorationis S12(T) | 99.85 | 89.4 | AXZL01000039 |
| SRB63 | 8-S-1 | Pseudoalteromonas lipolytica LMEB 39(T) | 100 | 89.5 | FJ404721 |
| SRB64 | 8-S-2 | Pseudoalteromonas lipolytica LMEB 39(T) | 98.85 | 89.5 | FJ404721 |
| SRB65 | 8-S-3 | Marinobacter lipolyticus SM19(T) | 98.09 | 89.6 | ASAD01000031 |
| SRB66 | 8-S-5 | Pseudoalteromonas lipolytica LMEB 39(T) | 100 | 89.5 | FJ404721 |
| SRB67 | 8-S-6 | Marinomonas rhizomae IVIA-Po-145(T) | 97.93 | 89.6 | EU188443 |
| SRB68 | 8-S-7 | Marinomonas rhizomae IVIA-Po-145(T) | 97.86 | 89.6 | EU188443 |
| SRB69 | 8-S-8 | Marinomonas rhizomae IVIA-Po-145(T) | 97.93 | 89.6 | EU188443 |
| SRB70 | 8-S-9 | Marinomonas rhizomae IVIA-Po-145(T) | 97.78 | 89.5 | EU188443 |
| SRB71 | 8-S-10 | Pseudoalteromonas lipolytica LMEB 39(T) | 100 | 89.5 | FJ404721 |
| SRB72 | 8-S-11 | Marinomonas rhizomae IVIA-Po-145(T) | 97.86 | 89.5 | EU188443 |
| SRB73 | 8-S-12 | Pseudoalteromonas lipolytica LMEB 39(T) | 99.92 | 89.5 | FJ404721 |
| SRB74 | 8-S-13 | Pseudoalteromonas lipolytica LMEB 39(T) | 100 | 89.5 | FJ404721 |
| SRB75 | 8-S-14 | Pseudoalteromonas lipolytica LMEB 39(T) | 100 | 89.5 | FJ404721 |
| SRB76 | 8-S-15 | Pseudomonas pachastrellae KMM 330(T) | 98.4 | 89.8 | AB125366 |
| SRB77 | 8-S-16 | Pseudoalteromonas lipolytica LMEB 39(T) | 100 | 89.5 | FJ404721 |
| SRB78 | 8-S-17 | Marinomonas rhizomae IVIA-Po-145(T) | 97.7 | 89.4 | EU188443 |
| SRB79 | 8-S-18 | Marinomonas rhizomae IVIA-Po-145(T) | 97.78 | 89.6 | EU188443 |
| SRB80 | 8-S-19 | Pseudoalteromonas lipolytica LMEB 39(T) | 100 | 89.5 | FJ404721 |
| SRB81 | 8-S-20 | Pseudoalteromonas lipolytica LMEB 39(T) | 100 | 89.5 | FJ404721 |
| SRB82 | 8-S-22 | Pseudoalteromonas lipolytica LMEB 39(T) | 100 | 89.5 | FJ404721 |
| SRB83 | 8-S-23 | Idiomarina maritima 908087(T) | 98.24 | 89.4 | EU600203 |
| SRB84 | 8-S-24 | Pseudoalteromonas lipolytica LMEB 39(T) | 99.62 | 89.6 | FJ404721 |
| SRB85 | 8-S-25 | Pseudoalteromonas lipolytica LMEB 39(T) | 100 | 89.5 | FJ404721 |
| SRB86 | 8-S-26 | Marinomonas rhizomae IVIA-Po-145(T) | 97.86 | 89.5 | EU188443 |
| SRB87 | 8-S-27 | Pseudoalteromonas lipolytica LMEB 39(T) | 100 | 89.5 | FJ404721 |
| SRB88 | 8-S-28 | Pseudoalteromonas lipolytica LMEB 39(T) | 100 | 89.5 | FJ404721 |
| SRB89 | 8-S-29 | Pseudoalteromonas lipolytica LMEB 39(T) | 100 | 89.5 | FJ404721 |
| SRB90 | 8-S-30 | Marinobacterium georgiense KW-40(T) | 99.85 | 89.4 | U58339 |
| SRB91 | 8-S-31 | Marinomonas rhizomae IVIA-Po-145(T) | 97.63 | 89.6 | EU188443 |
| SRB92 | 8-S-32 | Marinomonas alcarazii IVIA-Po-14b(T) | 95.45 | 89.2 | EU188442 |
| SRB93 | 8-S-33 | Marinomonas rhizomae IVIA-Po-145(T) | 97.78 | 89.6 | EU188443 |
| SRB94 | 8-S-34 | Marinomonas rhizomae IVIA-Po-145(T) | 97.55 | 89.6 | EU188443 |
| SRB95 | 9-S-1 | 'Acinetobacter bohemicus' ANC 3994 | 96.72 | 89.7 | KB849175 |
| SRB96 | 9-S-2 | Morganella morganii subsp. sibonii DSM 14850(T) | 99.07 | 89.3 | DQ358146 |
| SRB97 | 9-S-3 | Pseudomonas chengduensis MBR(T) | 99.92 | 89.8 | EU307111 |
| SRB98 | 9-S-4 | Pseudomonas chengduensis MBR(T) | 99.92 | 89.8 | EU307111 |
| SRB99 | 9-S-5 | Microbacterium lacus A5E-52(T) | 98.63 | 90.7 | AB286030 |
| SRB100 | 9-S-7 | Chryseobacterium taeanense PHA3-4(T) | 99.85 | 91 | AY883416 |
| SRB101 | 15-S-1 | Acinetobacter towneri DSM 14962(T) | 99.54 | 89.7 | APPY01000064 |
| SRB102 | 15-S-2 | Microbacterium flavum YM18-098(T) | 98.32 | 90.7 | AB286029 |
| SRB103 | 15-S-3 | Pseudomonas peli R-20805(T) | 98.78 | 89.7 | AM114534 |
| SRB104 | 15-S-4 | Pseudomonas peli R-20805(T) | 98.78 | 89.8 | AM114534 |
| SRB105 | 15-S-5 | Dietzia maris DSM 43672(T) | 99.77 | 91 | X79290 |
| SRB106 | 9-S-8 | Chryseobacterium taeanense PHA3-4(T) | 99.77 | 91 | AY883416 |
| SRB107 | 9-S-12 | Candidatus Devosia euplotis LIV5 | 98.09 | 93.3 | AJ548825 |
| SRB108 | 9-S-13 | Pseudomonas pachastrellae KMM 330(T) | 98.55 | 89.9 | AB125366 |
| SRB109 | 9-S-14 | Erythrobacter gangjinensis K7-2(T) | 96.64 | 93.2 | EU428782 |
| SRB110 | 9-S-22 | Roseovarius crassostreae CV919-312(T) | 99.62 | 94.2 | AF114484 |
| SRB111 | 15-S-6 | Aeromonas hydrophila subsp. hydrophila ATCC 7966(T) | 99.85 | 89.3 | CP000462 |
| SRB112 | 15-S-7 | Corynebacterium variabile DSM 20132(T) | 99.24 | 90.3 | AJ222815 |
| SRB113 | 15-S-8 | Bacillus flexus IFO 15715(T) | 99.85 | 88.8 | AB021185 |
| SRB114 | 15-S-9 | 'Deinococcus radiotolerans' C1(T) | 98.24 | 91.9 | KC771028 |
| SRB115 | 15-S-11 | Bacillus cereus ATCC 14579(T) | 99.92 | 88.9 | AE016877 |
| SRB116 | 15-S-12 | Dietzia maris DSM 43672(T) | 99.77 | 91.2 | X79290 |
| SRB117 | 15-S-13 | Bacillus anthracis ATCC 14578(T) | 99.92 | 88.9 | AB190217 |
| SRB118 | 15-S-14 | Halomonas meridiana DSM 5425(T) | 99.24 | 90 | AJ306891 |
| SRB119 | 15-S-16 | Roseovarius crassostreae CV919-312(T) | 99.77 | 94.2 | AF114484 |
| SRB120 | 15-S-18 | Pseudomonas peli R-20805(T) | 98.78 | 89.8 | AM114534 |
| SRB121 | 15-S-19 | Pseudomonas peli R-20805(T) | 98.7 | 89.8 | AM114534 |
